# Presence of Vaccine-Derived Newcastle Disease Viruses in Wild Birds

**DOI:** 10.1371/journal.pone.0162484

**Published:** 2016-09-14

**Authors:** Andrea J. Ayala, Kiril M. Dimitrov, Cassidy R. Becker, Iryna V. Goraichuk, Clarice W. Arns, Vitaly I. Bolotin, Helena L. Ferreira, Anton P. Gerilovych, Gabriela V. Goujgoulova, Matheus C. Martini, Denys V. Muzyka, Maria A. Orsi, Guilherme P. Scagion, Renata K. Silva, Olexii S. Solodiankin, Boris T. Stegniy, Patti J. Miller, Claudio L. Afonso

**Affiliations:** 1 College of Veterinary Medicine, University of Georgia, Athens, Georgia, United States of America; 2 Exotic and Emerging Avian Viral Diseases Research Unit, Southeast Poultry Research Laboratory, United States National Poultry Research Center, Agricultural Research Service, United States Department of Agriculture, Athens, Georgia, United States of America; 3 National Diagnostic Research Veterinary Medical Institute, Sofia, Bulgaria; 4 Odum School of Ecology, University of Georgia, Athens, Georgia, United States of America; 5 National Scientific Center Institute of Experimental and Clinical Veterinary Medicine, Kharkiv, Ukraine; 6 Laboratory of Animal Virology, Institute of Biology, University of Campinas-UNICAMP, Campinas, Brazil; 7 Department of Veterinary Medicine, College of Animal Science and Food Engineering and Graduate Program in Experimental Epidemiology of Zoonosis, University of São Paulo, São Paulo, Brazil; 8 Post-Graduate Program in the Experimental Epidemiology of Zoonoses, School of Veterinary Medicine and Animal Science, University of São Paulo, São Paulo, Brazil; 9 National Agricultural Laboratory of São Paulo, Lanagro/SP, Campinas, Brazil; Linnaeus University, SWEDEN

## Abstract

Our study demonstrates the repeated isolation of vaccine-derived Newcastle disease viruses from different species of wild birds across four continents from 1997 through 2014. The data indicate that at least 17 species from ten avian orders occupying different habitats excrete vaccine-derived Newcastle disease viruses. The most frequently reported isolates were detected among individuals in the order *Columbiformes* (n = 23), followed in frequency by the order *Anseriformes* (n = 13). Samples were isolated from both free-ranging (n = 47) and wild birds kept in captivity (n = 7). The number of recovered vaccine-derived viruses corresponded with the most widely utilized vaccines, LaSota (n = 28) and Hitchner B1 (n = 19). Other detected vaccine-derived viruses resembled the PHY-LMV2 and V4 vaccines, with five and two cases, respectively. These results and the ubiquitous and synanthropic nature of wild pigeons highlight their potential role as indicator species for the presence of Newcastle disease virus of low virulence in the environment. The reverse spillover of live agents from domestic animals to wildlife as a result of the expansion of livestock industries employing massive amounts of live virus vaccines represent an underappreciated and poorly studied effect of human activity on wildlife.

## Introduction

The livestock-wildlife interface, historically underappreciated as a cause of disease emergence, is now recognized as an intersection from which pathogens can be transmitted from agricultural to free-ranging hosts, and vice versa [1–4]. Some factors responsible for the presence of etiological agents at this interface include ecological changes such as spatial and temporal land-use alterations, pathogen adaptations to new hosts, and the introduction of non-native, permissive species [5–8]. Of all the factors influencing disease emergence, likely the most substantial is the loss of ecological species barriers, permitting opportunistic pathogens access to wildlife [9–13].

Microbial bidirectional spillover between wildlife and domesticated species is a recognized but understudied event [10, 14, 15]. As an example, avian influenza is a biosecurity threat to the poultry industry due to its spillover from waterfowl reservoirs and subsequent viral spill back from domestic chickens (*Gallus gallus*) [16, 17]. What remains largely unstudied, however, is the spillover of live vaccines from domesticated species into wildlife. For the purpose of this paper we define “spillover or spillover of live vaccines” as the unintended transmission of live poultry vaccine viruses from domestic, gallinaceous poultry into non-target birds. Inadequate recognition of spillover events may be due to a lack of surveillance efforts, low mortality of infected animals, migration, or undetected resident wild bird morbidity [18–20]. The continuous expansion of the poultry industry, coupled with the mass employment of live virus vaccines, increases the probability of spillover of vaccines [21–23].

Examining the extent of spillover of live vaccines, including recently developed recombinant vaccines, from poultry into wild birds is crucial because the downstream epidemiological consequences of such spillovers are still unknown. Poultry producers routinely employ multiple live vaccines against economically significant pathogens, such as Marek’s disease virus, Infectious bursal disease virus, Infectious bronchitis virus, Infectious laryngotracheitis virus, and Newcastle disease virus (NDV) [24–27]. Circulating live vaccine viruses present additional risks such as reversion of virulence and recombination with wild-type strains. In addition, the immune response of wild birds induced by infection with vaccine strains may provide selective pressures resulting in viral antigenic drift or increased virulence [23, 28–31].

Nonetheless, the use of live vaccines continues to dominate the poultry industry, as attested by their performance in the field. Their use is highly desirable as they are inexpensive, allow mass application, and stimulate both strong mucosal and cell-mediated immunity [32–34]. In the United States, the continuous demand for more effective vaccines is also likely driven by the industry’s prior experience with large-scale poultry epidemics. For example, producers in California experienced Newcastle disease (ND) outbreaks in 1971 and again in 2002, each costing millions of dollars to eradicate and requiring the depopulation of millions of birds [35, 36].

Newcastle disease virus, also known as Avian paramyxovirus-1 (APMV-1), is a non-segmented, single-stranded RNA virus of the genus *Avulavirus* within the family *Paramyxoviridae* [37]. In particular, live vaccines against ND, a disease notifiable to the World Organization for Animal Health (OIE), are used both intensively and globally [38, 39]. The world poultry industry with tens of billions of commercial fowl is continuously expanding [40]. However, NDV also infect a broad range of free-ranging avifauna [41, 42], notably Double-crested Cormorants (*Phalacrocorax auritus*) [43], dabbling ducks such as Mallards (*Anas platyrynchos*) [44], and even Adélie Penguins (*Pygoscelis adeliae*) from the South Shetland Islands [45].

Considering all of the aforementioned facts and the preliminary evidence of the presence of live vaccines in wild birds [46, 47], we examined the hypothesis that NDV vaccines may spill into wild birds. First, we identified in GenBank databases existing cases of vaccine-derived NDV reported previously in wild birds [48]. Second, to expand upon those results, we also sequenced NDV wild bird isolates from Ukraine, Bulgaria and Brazil. Third, we performed active NDV surveillance in a wild Rock Pigeon (*Columba livia*) population within an urban environment (Atlanta, GA, USA) to assess the presence of Newcastle disease viruses. Lastly, we analyzed the compilation of the GenBank and newly obtained wild bird NDV sequences, and compared them to currently used live NDV vaccines.

## Materials and Methods

### Ethics Statement

The fieldwork was conducted under the Georgia Department of Natural Resources Permit Number 29—WJH—16–48, and was conducted according to University of Georgia Institutional Animal Care and Use Committee (IACUC) protocol AUP #: A2012 03-031-Y1-A0. All birds were captured on public land owned and managed by the City of Atlanta. No protected, endangered or threatened species were involved in this research, and Rock Pigeons captured in the summer were all released within thirty minutes of capture, as per permit regulations. Rock Pigeons captured by APHIS followed APHIS Wildlife Services protocols as outlined in the 2011 State of Georgia Environmental Assessment Protocols [49].

### Capture and Sampling of Rock Pigeons

Rock Pigeons (n = 72) were captured at three urban sites inside the city limits of Atlanta, Georgia (Fulton County) between May and October 2012 within a 10 km^2^ area. From May to July (summer capture), pigeons were caught at three urban sites, while from August to October 2012 (fall capture), pigeons were captured by the USDA-APHIS Wildlife Services personnel at multiple sites as part of their integrated wildlife damage management program [50]. Pigeons were captured using a combination of hand-nets, drop-nets, mist-nets, and ground traps. During the summer capture period, all individuals were banded with a unique four-color combination to avoid pseudoreplication [51].

To ensure consistency, assessment of demographic attributes, recording, and analysis of all wild Rock Pigeon physiological characteristics was performed by the same person. Each bird was placed into one of two age classes: hatch-year birds (HY) who were born during the current breeding cycle or after hatch-year birds (AHY) born prior to 2012 [52]. Breeding status was established using reproductive characteristics, such as the presence of a brood patch, cloacal protuberance, or both [53]. The sex of AHY birds was determined by reproductive characteristics (if present) and, when observed, by courtship behaviors [54]. Individual body conditions were assessed using two different criteria: the ratio of bird mass to wing chord length (also known as the wing-loading aspect), and the relative amount of visible fat. The weight-to-wing chord ratio was calculated as the body mass divided by the length of the un-flattened longest primary feather [53, 55], while the MAPS (Monitoring Avian Productivity and Survival) protocol [56] was used for fat scoring. Only wing-pit fat was assessed due to the density of plumage at the furculum and stomach, and scored on an eight-point scale of 0 to 7. A zero fat score indicated no visible yellow subcutaneous fat at the wing-pit and a score of seven indicated a bulging pocket of fat.

Up to 1 mL of blood was taken from the brachial vein of each individual, transferred to a 3 mL serum vacutainer (Becton Dickinson, San Jose, CA, USA), and placed in a cooler with ice packs for transport to the lab. Vacutainers were tilted overnight at room temperature, then centrifuged at 1500 rpm, and the harvested sera were subsequently stored at -20°C [57]. Oral and cloacal swabs were collected from each bird, then transferred into separate 2.0 mL cryovials (Corning Inc., Corning, NY, USA) containing 1.5 mL of Brain-Heart Infusion (Becton Dickinson, San Jose, CA, USA) mixed with Gentamicin (200 μg/mL), Penicillin (2000 units/mL), and Amphotericin-B (4 μg/mL). Cryovials containing swabs were chilled in a cooler with ice packs, transported to the lab and then stored at -80°C until processed [58].

### Rock Pigeon virus Titration, Intracerebral Pathogenicity Index (ICPI) Test, and Serology

Swab medium from each cryovial was inoculated in 9–11 day-old specific-pathogen-free (SPF) embryonated chicken eggs (ECE) in Biosafety Level-2 at the Southeast Poultry Research Laboratory (SEPRL). The recovered allantoic fluid was passaged once more. Protocols for virus isolation and titration followed OIE standards [59]. After a week of incubation, harvested allantoic fluids were tested using the hemagglutination assay (HA). Samples with HA activity by the second passage were considered positive and set aside for further characterization. Viral titers were assessed via 1:10 serial dilutions (10^−5^ to 10^−10^). Five SPF ECE per dilution were inoculated with 100 μL of each dilution, incubated for a week, then evaluated with the HA test. The viral mean embryo infectious dose (EID_50_) were calculated as described previously [60].

The virulence of Rock Pigeon isolates was determined from allantoic fluid by ICPI test in 1-day-old SPF chickens [61]. Viruses with ICPI values below 0.7 were considered of low virulence [59]. Lastly, Rock Pigeon sera were individually assessed using hemagglutination inhibition (HI) assay in 96-well plates [59] using antigens for APMV serotypes 1, 2, 3, 4, 6, and 7 [62]. Only samples demonstrating complete HI at the serum dilution 1:16 (2^4^) or higher were considered positive for presence of NDV antibodies [63].

### RNA Extraction, PCR Amplification and Sequencing

Newcastle disease viruses from laboratory repositories in Bulgaria and Ukraine (Table 1) were submitted to SEPRL for further characterization. Each sample was propagated into 9–11 day-old SPF ECE using standard methods [59]. RNA extractions of USA pigeon isolates and viruses from Bulgaria and Ukraine were performed using TRIzol LS (Life Technologies, Carlsbad, CA, USA), as per manufacturer instructions. One-step reverse-transcriptase PCR amplification proceeded using the SuperScript^®^ III One-Step RT-PCR System with Platinum^®^ *Taq* DNA Polymerase (Life Technologies, Carlsbad, CA, USA) and previously described primers (4331F/5090R, 4911F/5857R, 5669F/6433R, 4961F/5772R) were used for the PCR and sequencing [64]. Amplicons were separated through a 1% agarose gel, with the ensuing DNA bands excised and purified using the QuickClean II Gel Extraction Kit (GenScript, Piscataway, NJ, USA). Nucleotide sequencing and assembly were performed as described previously [39].

**Table 1:** Collated Isolates from GenBank and SEPRL samples. A total of 54 isolates from the following taxonomic orders are tabulated below: *Accipitriformes* (n = 1); *Anseriformes* (n = 13); *Charadriiformes* (n = 3); *Columbiformes* (n = 23); *Falconiformes* (n = 1); *Galliformes* (n = 4); *Passeriformes* (n = 2); *Pelecaniformes* (n = 1); *Phoenicopteriformes* (n = 1); *Psittaciformes* (n = 4); Unknown (n = 1). GenBank accession numbers bolded are strains sequenced from this study.

GenBank Accession Number	Genotype	Vaccine type	Order (Host)	Country/Province	Isolate	Year of isolation	Reference
^a^**KU133357**	II	B1	Columbiformes (Rock Pigeon)	Brazil/Sao Paulo State	8705	2009	This Study
^a^**KU133359**	II	B1	Columbiformes (Rock Pigeon)	Brazil/Sao Paulo State	S67A	2009	This Study
^a^**KU133356**	II	B1	Falconiformes (Peregrine Falcon)	Brazil/Sao Paulo State	PET26711	2009	This Study
^b^**KU133361**	II	B1	Psittaciformes (Vinaceous-breasted Amazon)	Brazil/Santa Catarina State	S331	2011	This Study
**KU133360**	II	B1	Anseriformes (Black Swan)	Brazil/Sao Paulo State	A07	2013	This Study
**KU133358**	II	B1	Columbiformes (Rock Pigeon)	Brazil/Rio Grande do Sul State	UFRGS	2014	This Study
FJ480821.1	II	B1	Anseriformes (Goose)	China/Fujian	FJ/1	2006	[48]
FJ480823.1	II	B1	Anseriformes (Goose)	China/Heilongjiang	HLJ/2	2006	[48]
**KU159667**	II	B1	Columbiformes (Rock Pigeon)	USA/Georgia	GA/1	2012	This Study
**KU159668**	II	B1	Columbiformes (Rock Pigeon)	USA/Georgia	GA/2	2012	This Study
**KU159669**	II	B1	Columbiformes (Rock Pigeon)	USA/Georgia	GA/3	2012	This Study
**KU159670**	II	B1	Columbiformes (Rock Pigeon)	USA/Georgia	GA/4	2012	This Study
**KU159671**	II	B1	Columbiformes (Rock Pigeon)	USA/Georgia	GA/5	2012	This Study
**KU159672**	II	B1	Columbiformes (Rock Pigeon)	USA/Georgia	GA/6	2012	This Study
**KU159673**	II	B1	Columbiformes (Rock Pigeon)	USA/Georgia	GA/7	2012	This Study
**KU159674**	II	B1	Columbiformes (Rock Pigeon)	USA/Georgia	GA/8	2012	This Study
**KU159675**	II	B1	Columbiformes (Rock Pigeon)	USA/Georgia	GA/9	2012	This Study
JX901331.1	II	B1	Columbiformes (Pigeon)	USA/Pennsylvania	0702.2	2007	[111]
JX901343.1	II	B1	Columbiformes (Pigeon)	USA/Minnesota	0713.2	2007	[111]
^b^AY727883.1	II	LaSota	Phoenicopteriformes (Chilean Flamingo)	Argentina/Buenos Aires	88T.00	2000	[112]
**KU133355**	II	LaSota	Columbiformes (Rock Pigeon)	Bulgaria/Sofia	Govedartzi	2002	This Study
**KU133351**	II	LaSota	Unknown	Bulgaria/Varna	Varna	2006	This Study
**KU133352**	II	LaSota	Anseriformes (Mute Swan)	Bulgaria/Burgas	Ravna Gora	2006	This Study
**KU133353**	II	LaSota	Anseriformes (Mute Swan)	Bulgaria/Burgas	Malko Tarnovo	2006	This Study
**KU133354**	II	LaSota	Anseriformes (Mallard Hybrid)	Bulgaria/Haskovo	Simeonovgrad	2007	This Study
AF079324.1	II	LaSota	Columbiformes (Pigeon)	China/Heilongjiang	PNDV1	1998	[48]
DQ363532.1	II	LaSota	Anseriformes (Goose)	China/Jiangsu	Jiangsu/JS05	2003	[113]
FJ938171.1	II	LaSota	Passeriformes (House Sparrow)	China/Guangxi	Guangxi/NN10	2007	[114]
DQ228923.1	II	LaSota	Anseriformes (Duck)	China/Shangdong	Shandong/SY	2003	[113]
JX244804.1	II	LaSota	Columbiformes (Pigeon)	China/Guangdong	111	2008	[48]
JX244807.1	II	LaSota	Columbiformes (Pigeon)	China/Guangdong	114	2008	[48]
JX193080.1	II	LaSota	Anseriformes (Duck)	China/Guangxi	Guangxi19	2009	[48]
JX193082.1	II	LaSota	Anseriformes(Duck)	China/Guangxi	Guangxi21	2010	[48]
JX482548.1	II	LaSota	Charadriiformes (Gull/Tern)	China/Shangdong	H1 (SD1 in paper)	2011	[115]
JX482549.1	II	LaSota	Charadriiformes (Shorebird/Wader)	China/Jiangsu	H5 (JS1 in paper)	2011	[115]
JX482551.1	II	LaSota	Charadriiformes (Shorebird/Wader)	China/Guangdong	H37 (GD1 in paper)	2011	[115]
KM670001.1	II	LaSota	Anseriformes (Duck)	China/Shangdong	SD09	2014	[48]
^b^AY359876.1	II	LaSota	Psittaciformes(Psittacine)	India	NDVCUL	1997	[48]
KC808487.1	II	LaSota	Passeriformes (Montezuma Oropendola)	Mexico/Chiapas	Chiapas/661-ZM01	2008	[47]
^c^KC808489.1	II	LaSota	Accipitriformes (Short-tailed Hawk)	Mexico/Chiapas	Chiapas/663-ZM03	2008	[47]
^b^^,^^c^KC808490.1	II	LaSota	Psittaciformes (Yellow-naped Parrot)	Mexico/Chiapas	Chiapas/664-ZM04	2008	[47]
KC808497.1	II	LaSota	Pelecaniformes (Great Egret)	Mexico/Chiapas	Chiapas/671-ZM11	2009	[47]
**KU133362**	II	LaSota	Columbiformes (Pigeon)	Ukraine/Donetsk	Donetsk/3	2007	This Study
**KU133363**	II	LaSota	Columbiformes (Pigeon)	Ukraine/Dnipropetrovsk	7/Dnipropetrovsk	2007	This Study
**KU133364**	II	LaSota	Columbiformes (Pigeon)	Ukraine/Kharkiv	Kharkiv/1	2007	This Study
**KU133365**	II	LaSota	Columbiformes (Pigeon)	Ukraine/Kharkiv	Kharkiv/2	2007	This Study
JX901345.1	II	LaSota	Columbiformes (Pigeon)	USA/Maryland	0715	2007	[111]
JX193079.1	Ia	V4	Anseriformes (Duck)	China/Guangxi	Guangxi18	2009	[48]
KP001164.1	Ia	V4	Anseriformes (Duck)	China/Heilongjiang	HLJ/0214-2	2011	[48]
^b^KC808493.1	Ic	PHY-LMV42	Psittaciformes (Red-lored Amazon Parrot)	Mexico/Chiapas	Chiapas/667-ZM07	2009	[47]
KC808494.1	Ic	PHY-LMV42	Galliformes (Plain Chachalaca)	Mexico/Chiapas	Chiapas/668-ZM08	2009	[47]
KC808495.1	Ic	PHY-LMV42	Galliformes (Plain Chachalaca)	Mexico/Chiapas	Chiapas/669-ZM09	2009	[47]
KC808496.1	Ic	PHY-LMV42	Galliformes (Plain Chachalaca)	Mexico/Chiapas	Chiapas/670-ZM10	2009	[47]
^b^^,^^c^KC808498.1	Ic	PHY-LMV42	Galliformes (Highland Guan)	Mexico/Chiapas	Chiapas/674-ZM14	2009	[47]

^a^birds in captivity.

^b^endangered species as determined by the International Conservation Union for Nature (IUCN) in sampled regions.

^c^zoo birds.

The Brazilian samples were characterized at the University of São Paulo (Brazil). Viral RNA purifications were performed with QIAamp Viral RNA Mini Kit (Qiagen, Hilden, Germany), according to manufacturer’s instructions, followed by RT-PCR using SuperScript^®^ III One-Step RT-PCR System with Platinum^®^ *Taq* DNA Polymerase (Life Technologies, Carlsbad, CA, USA) and previously described primers [64]. Amplicons were visualized in 2% of SYBR Safe (Life Technologies, Carlsbad, CA, USA) low Melting Point Agarose (Life Technologies, Carlsbad, CA, USA). Products were purified using Illustra GFX PCR DNA and Gel Band Purification Kits (GE Health Care and Life Sciences, Buckinghamshire, England). DNA sequencing was performed with BigDye Terminator v3.1 Cycle Sequencing Kit (Life Technologies, Carlsbad, CA, USA) in ABI 3730XL DNA Analyzer (Applied Biosystems, Foster City, CA USA) at the LACTAD Facility at UNICAMP (http://www.lactad.unicamp.br/en).

### GenBank Sequence Compilation and Phylogenetic Analyses

All available complete fusion-gene (F-gene) sequences of class II NDV were downloaded from GenBank as of July 2015 [48] and aligned using ClustalW [65], resulting in 1452 complete fusion protein gene sequences. Initial phylogenetic analyses were performed utilizing the complete F-gene sequences using the Neighbor Joining method based upon 100 bootstrap replicates [66], as implemented in the MEGA version 6 software [67] (data not shown). To ensure that all viruses used in further analyses were isolated only from wild birds, we subjected the dataset to rigorous selection criteria. Specifically, viruses isolated from domestic non-poultry species such as waterfowl from live bird markets [68], and sporting, racing, or pet birds where conspecifics may have been vaccinated [69] were excluded from the dataset. Viral sequences of wild bird isolates (n = 54, including the 24 sequenced in this study) (Table 1) that were evolutionarily closely related to reference vaccine strains (n = 5) from genotypes I and II were parsed from the initial compilation. An additional 15 representative sequences from the remaining genotypes (III-XIV and XVI-XVIII) were also included, resulting in a final dataset of 74 sequences.

Analysis of the best-fit substitution model was performed and the goodness-of-fit for each model was measured by the corrected Akaike Information Criterion (AICc) and the Bayesian Information Criterion (BIC) [67]. Tamura 3-parameter model with 500 bootstrap replicates was used for constructing the phylogenetic tree [70]. Finally, estimates of the means and pairwise genetic distances were computed using the Maximum Composite Likelihood method as implemented in MEGA6 [71]. The pairwise distances per decade were also calculated as the nucleotide distance per site divided by the number of years separating the isolation of each virus and the respective vaccine strain, multiplied by ten**.** The rate variation among sites was modeled with a gamma distribution (shape parameter = 4). For each statistical inference, codon positions consisting of 1st+2nd+3rd+Noncoding were retained, while gaps and/or missing data were trimmed.

In the past, both topology and nucleotide distance have been used to demonstrate that the sources (direct or indirect) of isolated viruses were live vaccines used in poultry [38, 46, 72]. It has been previously reported that a nucleotide distance of approximately 1% per decade is the natural rate of NDV evolution [73, 74]. In the present study, more stringent selection criterion to identify vaccine-derived viruses was used. NDV isolates with nucleotide distances lower than 0.1% per decade, when compared to the most closely related vaccine strains, were termed “vaccine-derived” viruses.

### Statistical Analyses

All statistical analyses were performed with SAS v. 9.3 [75]. Fisher’s Exact Test for small sample sizes and dichotomous variables [76] (a 2 x 2 contingency table) was used to examine whether a significant relationship existed among the ages at which NDV infected Rock Pigeons were found shedding virus. For that purpose, the covariates age and shedding status were each divided into two classes, the hatch-year (HY) and after hatch-year (AHY), and shedding or not shedding, respectively. We next investigated whether a measurable physiological cost was present in the wild Rock Pigeons infected with vaccine-derived NDV. We used two widely accepted and correlated measures of body condition indices in passerines and near passerines to do so: i) weight-to-wing chord ratio, which is measured on a continuous range, and ii) fat scores, which are measured along an ordinal scale [77]. Prior to analyses, the distributions for fat and weight-to-wing chord ratio were inspected to confirm that each variable met the assumption for a non-skewed distribution using the Shapiro Wilk’s test statistic [78].

Since the variable fat (S2 Table) did not meet the assumption of a normalized distribution, we utilized the non-parametric, one-way-ANOVA-by-ranks Kruskal-Wallis H test (S3 Table) [79] to determine whether the fat scores of shedding Rock Pigeons were significantly lower than the fat scores of non-shedding Rock Pigeons. While the variable weight-to-wing chord ratio had a normal distribution (S4 Table), it was necessary to relax assumptions regarding the within-group variances due to the unequal sample sizes of shedding Rock Pigeons versus non-shedding Rock Pigeons [80]. Therefore, we used a one-way generalized linear model (GLM) (S5 Table) to examine whether shedding Rock Pigeons had a measurably smaller weight-to-wing chord ratio than their non-shedding counterparts.

### GenBank Submission of New Isolates Generated by This Study

The complete F-gene sequences (n = 24) of NDV obtained in this study were submitted to GenBank and are available under the accession numbers from KU133351 to KU133365 and KU159667 to KU159675.

## Results

### Sample Collection from Rock Pigeons, Serological, Virological, and ICPI Tests

Cloacal and oral swabs (n = 72) and blood samples (n = 71) were collected from Rock Pigeons in Atlanta, Georgia. Rock Pigeon body condition indices, age, serological and virus shedding status are provided in S1 Table. Three birds were positive for NDV antibodies, equaling a serological study prevalence of 4.23%. No NDV was isolated from the serologically positive birds. Nine Rock Pigeon samples tested positive via HA for NDV shedding. None of the samples cross-reacted with the additional tested APMV serotypes (data not shown). The detected shedding of NDV from the Rock Pigeon samples suggests active replication (Table 2). ICPI data of the HA positive samples are presented in Table 2. The observed low ICPI values (0.03 to 0.34) indicated that the isolated viruses were of low virulence [59].

**Table 2:** Rock Pigeon virus isolation, HI antibody titer, and intracerebral pathogenicity indices (ICPIs). Field swabs collected from the oral and cloacal cavities positive for virus are listed with a + symbol, otherwise an N/A is listed. ICPI experimental infection swabs collected in the lab from the oral and cloacal cavities positive for virus are listed below with their corresponding ICPI values, otherwise an N/A is listed.

	Viral Isolation		ICPI		
Rock Pigeon Isolate	Oral Sample	Cloacal Sample	Oral Sample	Cloacal Sample	HI Antibody Titer
Rock Pigeon/USA/GA/1/2012	+	+	0.05	0.03	<2
Rock Pigeon/USA/GA/2/2012	N/A	+	N/A	0.11	<2
Rock Pigeon/USA/GA/3/2012	+	+	0.03	0.14	<2
Rock Pigeon/USA/GA/4/2012	+	+	0.04	0.05	<2
Rock Pigeon/USA/GA/5/2012	+	+	0.11	0.30	<2
Rock Pigeon/USA/GA/6/2012	+	N/A	0.26	N/A	<2
Rock Pigeon/USA/GA/7/2012	+	+	0.05	0.34	<2
Rock Pigeon/USA/GA/8/2012	+	N/A	0.1	N/A	<2
Rock Pigeon/USA/GA/9/2012	+	N/A	0.03	N/A	<2

### Sequencing Results and Phylogenetic Analyses

**Fig 1:**
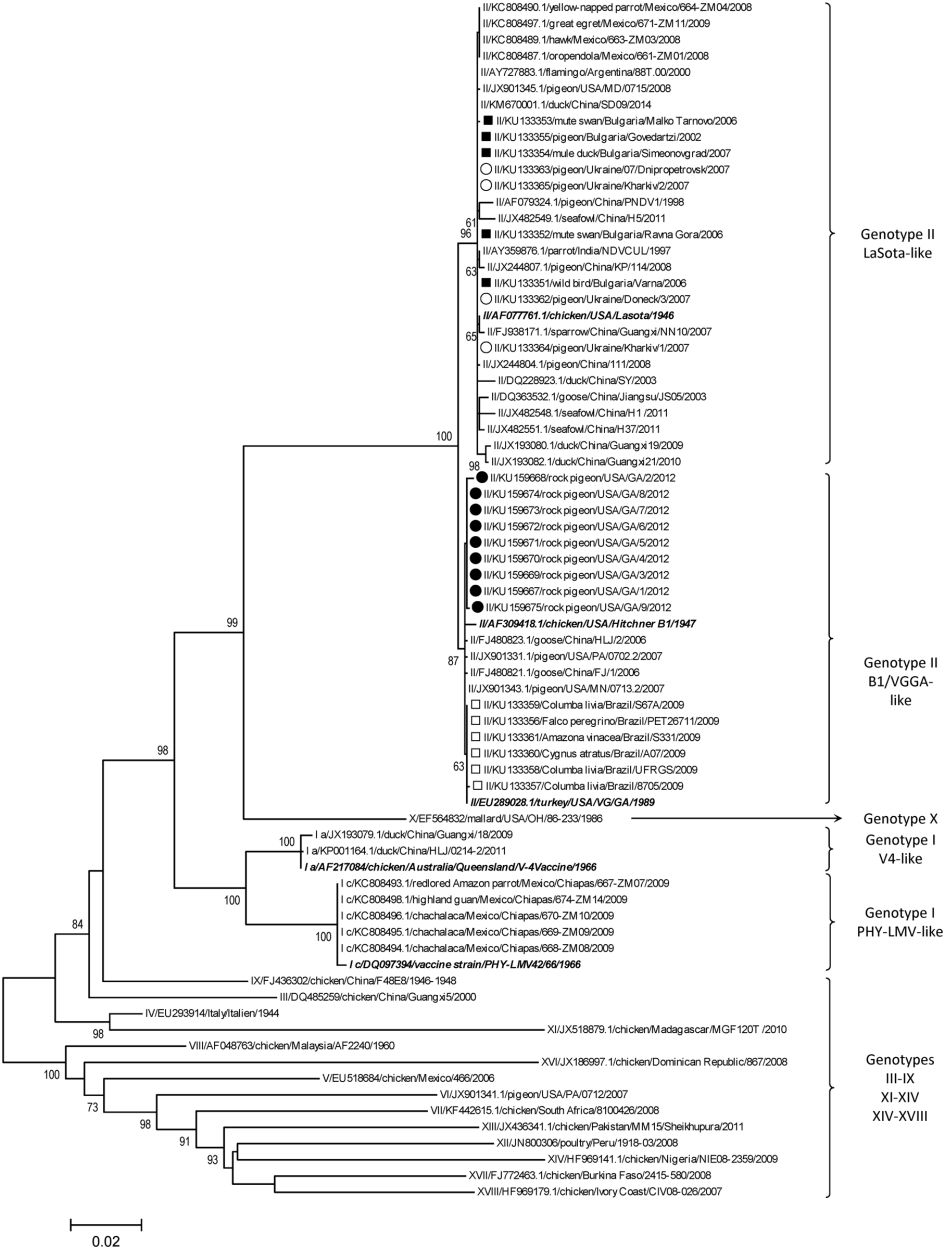
Phylogenetic tree of isolates and their relationship to class II NDV viruses. Phylogenetic analysis based on the complete nucleotide sequence of the fusion gene of isolates representing NDV class II. The evolutionary history was inferred by using the Maximum Likelihood method based on Tamura 3-parameter model with 500 bootstrap replicates [70]. The tree with the highest log likelihood (-108983.3717) is shown. A discrete Gamma distribution was used to model evolutionary rate differences among sites (4 categories (+G, parameter = 0.0936). The rate variation model allowed for some sites to be evolutionarily invariable ([+I], 39.7777% sites). The tree is drawn to scale with branch lengths measured in the number of substitutions per site and the percentage of trees in which the associated taxa clustered together are shown below the branches. The analysis involved 81 nucleotide sequences with a total of 1662 positions in the final dataset. Isolates studied in this work are designated in front of the taxa name as follows: USA—●; Ukraine—○; Brazil—□, Bulgaria—■. Evolutionary analyses were conducted in MEGA6 [67]. The Roman numerals presented in the taxa names in the phylogenetic trees represent the respective genotype for each isolate, followed by the GenBank identification number, host name (if available), country of isolation, strain designation and country of isolation.

Sequencing data from the F-gene analysis showed that the 24 isolates from wild birds in Brazil, Bulgaria, Ukraine and the USA contained fusion protein cleavage sites specific for NDV of low virulence with two basic amino acids between residue positions 113 and 116 and a leucine at position 117 (_113_RQGR↓L_117_) [59]. As a result of the preliminary phylogenetic analysis including all available sequences from GenBank and the 24 sequences obtained in this study (data not shown), 54 viral sequences that were evolutionarily closely related to reference vaccine strains from genotypes I and II were selected for further analysis. The corresponding data about these selected viruses are presented in Table 1. Utilizing established criteria, the 24 NDV sequences were classified as members of genotype II of class II [81]. Nine of them clustered together with the vaccine strain chicken/USA/LaSota/1946, while the other 15 isolates grouped with two additional ND vaccine strains of genotype II—chicken/USA/HitchnerB1/1947 and turkey/USA/VG/GA/1989 (Fig 1).

Fig 1 illustrates the distribution of the isolates from wild birds that are genetically closely related to vaccine strains of NDV genotype I and II. Within genotype II, 28 isolates from wild birds originating from Argentina, Bulgaria, China, India, Mexico and Ukraine clustered in a monophyletic branch with the vaccine strain chicken/USA/LaSota/1946. The mean genetic distance between this vaccine strain and the isolates within the branch was considerably low (0.2%). Within the same genotype, another 19 wild bird isolates from Brazil, China and USA grouped together with vaccine strains chicken/USA/HitchnerB1/1947 and turkey/USA/VG/GA/1989. The genetic analysis showed that the latter isolates of wild-bird origin displayed genetic distance of 0.3–0.4% when compared to these two vaccine strains. Similarly, seven wild-bird NDV isolates of genotype I of class II also showed close phylogenetic relationship with vaccine strains. Five Mexican isolates of wild-bird origin from 2009 clustered with the vaccine strain PHY-LMV42/1966, while two 2009 and 2011 Chinese isolates of duck origin grouped closely together in the phylogram with the vaccine strain chicken/Australia/Queensland/V-4/1966 (Fig 1). The mean genetic distance between the viruses and the vaccine strains within each of these genotype I branches was 0.2%. Pairwise distance and pairwise distance per decade results among viruses within of the described groups are presented in S7, S8 and S9 Tables. Based on the above results and the pairwise distances per decade (from 0.009% to 0.093%), the 54 viruses from these groups were determined as “vaccine-derived”.

Our data indicate that at least 17 species from ten avian orders occupying different habitats excreted vaccine-derived NDV (Table 1). The most frequent isolations occurred in the orders *Columbiformes* (n = 23) and *Anseriformes* (n = 13), with occasional isolations from eight other orders. The oldest isolation detected occurred in 1997 and the most recent in 2014 with samples from free-ranging (n = 47) and captive (n = 7) birds. The obtained results demonstrate that vaccine-derived NDV were detected in eight countries on four different continents. Lastly, some of the vaccine-derived viruses were obtained from wild birds representing declining species or species at imminent risk of decline according to international standards and conservation working groups (Table 1). These species of special concern include the Highland Guan (*Penelopina nigra*), Red-lored Amazon (*Amazona autumnalis*), Yellow-naped Amazon (*Amazona auropalliata*), Vinaceous-breasted Amazon (*Amazona vinacea*), Chilean Flamingo (*Phoenicopterus chilensis*), and an endemic Psittacine from India, a country where all Psittacines are facing declines [82].

### Statistical Analyses of Rock Pigeon Data

A total of 70 Rock Pigeons were assessed in quantifying whether age served as an explanatory variable for infection with vaccine-derived NDV (as the age of two captured Rock Pigeons could not be determined). Fisher’s Exact Test for small sample sizes and dichotomous variables was employed to determine whether the Rock Pigeon “age class” was a significant predictor of NDV infection. The results (Fisher’s Exact Test: *p* = 0.0217) provide evidence that HY birds are statistically more susceptible to infection with the vaccine-derived NDV in this population (S6 Table).

We hypothesized that Rock Pigeons infected with the vaccine-derived viruses would fall into a poorer body condition class than their non-shedding conspecifics. Only the fat scores and the weight-to-wing chord ratio values of HY birds were used in these analyses as only HY birds were infected with vaccine-derived viruses. HI titers were not added as a covariate to these analyses since all shedding birds were negative for NDV antibodies. We initially proposed that shedding HY birds would have a lower mean ranked fat score than non-shedding HY birds. We determined that there was no statistical difference in visible subcutaneous fat between shedding (n = 9) and non-shedding (n = 36) HY individuals (Kruskal-Wallis, H = 0.1117, p = 0.7382) (S3 Table). The variable weight-to-wing chord ratio is a well-accepted metric of body condition that is also frequently applied in avian disease ecology field studies (55, 56). We found no statistical difference in the weight-to-wing chord ratio indices between shedding birds and non-shedding birds (F = 0.17, *df* = 1, 42, p = 0.6844) (S5 Table).

## Discussion

The unexpectedly low genetic distances per decade (from 0.009% to 0.093%) between the vaccine strains and those isolates studied here demonstrate the global presence spanning at least 18 years of vaccine-derived ND viruses in wild birds (S7, S8 and S9 Tables). Although our data do not allow for the identification of the direct sources of NDV infection for the studied wild birds, it is reasonable to suggest that these wild bird isolates originated from recent spillovers of live NDV vaccines, instead of representing strains that naturally circulate in these birds for the following reasons: i) if the newly isolated viruses had been naturally circulating and originated from vaccine strains (originally isolated in the 1940s and the 1960s) through natural evolution they would have changed significantly for the last 4 to 6 decades and would have presented much higher nucleotide distances than the ones determined in the pairwise distance analysis (S7, S8 and S9 Tables); ii) preservation of live NDV in the environment, unchanged for such a long period of time, is highly unlikely due to the thermal and biological lability of NDV [83]; and iii) for the majority of the isolates studied here that are almost genetically identical, there is no evidence of a direct epidemiological link, neither geographical nor temporal, between their isolations.

Spillover of NDV vaccines into wild birds reflects the most commonly used live NDV vaccines. Four different types of vaccine-derived viruses have been identified in at least 17 wild bird species from 10 different orders. The identification rates of LaSota- and B1-like viruses were substantially higher compared to the rest of the vaccines (28 and 19, respectively, out of a total of 54), corresponding with the most widely utilized vaccines, LaSota and Hitchner B1 [84–86]. In addition, LaSota, the most pathogenic of the commonly used NDV vaccines [87], is more likely to be shed in the environment. Spillovers of other NDV vaccines that are more limitedly employed were identified at lower rates (PHY-LMV42 and V4), or were not found at all (NDW, I-2, F, Clone-30, Ulster) [42, 59, 86].

The presence of vaccine-derived NDV in non-target species is likely to be underestimated in surveillance and passive diagnostic studies. For instance, the recovery of vaccine-derived viruses from samples submitted to diagnostic laboratories is usually neglected since only the identification of pathogenic or virulent isolates are a priority for animal health. Newcastle disease vaccines, although often ignored, may behave similarly to more widely studied vaccines. There are multiple live vaccines that are widely used in human and animal medicine that are documented as being shed from inoculated patients (varicella, vaccinia, polio, distemper) into the environment [88–91]. Considering that NDV has the capacity to infect at least 250 species of birds and all avian species are considered susceptible [42, 92], these vaccines have the potential to be easily transmitted across species undetected.

Although our results are preliminary, the high number of cases and the biological and behavioral characteristics of the order *Columbiformes* (comprising primarily Rock Pigeons) [93, 94] suggest that these birds have a high degree of association with vaccine-derived viruses, and that they may be used as sentinels for spillover of live NDV vaccines. Furthermore, the synanthropic nature of many birds from the order *Columbiformes*, as well as their worldwide distribution [95–97] and presence in anthropogenic affected areas, increase the probability for cross-host transmission of NDV between poultry and wild birds. The hatch-year cohort of Rock Pigeons (Atlanta, GA, USA) represented 100% of all incidences of identification of vaccine-derived viruses within that population. This may be associated with the higher vulnerability to disease of juveniles due to their increased movement requirements for foraging, in concert with naïve immune systems [98, 99]. In the studied dataset, the order *Anseriformes* also had a high incidence of shedding of vaccine-derived NDV. The high rate of detection of vaccine-derived NDV from this order in Asia may reflect a higher degree of contact between wild birds and domestic members of this order as a result of the larger populations of ducks and geese used as poultry in this continent.

Although the analyses of fat scores and weight-to-wing chord ratios among the studied wild Rock Pigeons did not demonstrate a negative physiological impact derived from infection with vaccine-derived NDV, the ecological effects have yet to be addressed in other species. Newcastle disease virus vaccines are substantially beneficial for the control of the disease, but they have also been documented to have some mild to moderate negative side effects in poultry [33, 100–102]. The lack of recognition of adverse events or insufficient sampling efforts in wild avifauna [18, 19] may be the reasons why reports of such side effects are absent in the literature. An additional outcome of this study is that species reported to shed vaccine-derived NDV are listed by the International Union for Conservation of Nature as taxa of conservation concern. Some of the vaccine-derived viruses were obtained from wild birds representing declining species or species at imminent risk of decline and the ecological implications of this finding are currently unknown (Table 1).

Our analyses did not utilize a random dataset, and the possibility of sampling bias cannot be discarded. It is noteworthy to recognize that some degree of reporting bias may have influenced our results, as we are limited to analyzing self-submitted data [103]. The nature of epidemiological source data implies that convenience samples are often all that are available, and can be valuable in cross-sectional, observational studies [104–106]. Molecular epidemiology meta-analyses with access to convenience datasets comprised of GenBank genomic-associated data are widespread and useful tools. For example, a limited dataset of self-reported isolates from GenBank (n = 97) was used to pinpoint the source and direction of spread for Methicillin-resistant Staphylococcus aureus within hospitals of Florida [107]. Sequences from convenience samples of *Leishmania infantum* from Brazil (n = 45) submitted to GenBank were evaluated to determine that no statistical association existed between the host species (canids or humans) and genetic variability within the causative agent [108]. The emergence of severe acute respiratory syndrome (SARS) within densely populated Hong Kong prompted a study in which the only available dataset was a series of convenience samples of GenBank isolates from recently acquired clinical cases from China (n = 168). The authors not only identified two co-circulating SARS viral clusters, but also identified the host travel method for the viral isolate detected in North America [109].

It is difficult to quantify the magnitude of reporting bias since it is unknown how many birds have historically been positive for vaccine-derived viruses, yet were not reported to GenBank. The analysis of 54 isolates is a small sample size from which to draw inferences about the potential impact of spillovers of NDV vaccines; however, the referenced studies did not indicate that vaccine viruses were specifically targeted for sampling as opposed to wild-type NDV, and it was concluded that each vaccine-derived virus was an incidental research discovery. We utilized a convenience sample to make a causal inference. In public health and epidemiology, causal inference and risk management often use the best available data to identify when intervention is feasible and necessary [110].

## Conclusion

Further studies are necessary to evaluate if transmission of these and other vaccines or infectious agents from poultry operations into free-ranging avifauna would have significant ecological impact.

## Supporting Information

S1 Table Demographic and correlated viral data of Rock Pigeons sampled in Atlanta, GA. (DOCX)

S2 Table Rank sum scores for the variable Fat classified by the variable shedding. (DOCX)

S3 Table Kruskal-Wallis H test. Non-parametric test for ordinal values (HY birds only).
(DOCX)

S4 Table Shapiro Wilk’s Test for Normality for the variable weight-to-wing chord ratio (HY birds only). (DOCX)

S5 Table GLM for the variable weight-to-wing chord ratio (HY birds only), shedding versus non-shedding birds. (DOCX)

S6 Table Fisher’s Exact Test Full Model for comparing shedding vs. non-shedding by “age class” of Rock Pigeon sampled in Atlanta, GA. (DOCX)

S7 Table Pairwise nucleotide distance analysis of vaccine-derived viruses to V-4 vaccine. (XLS)

S8 Table Pairwise nucleotide distance analysis of vaccine-derived viruses to PHY-LMV vaccine. (XLS)

S9 Table Pairwise nucleotide distance analysis of vaccine-derived viruses to LaSota and B-1 vaccines. (XLS)
