# Supplementary material for: Presence of Vaccine-Derived Newcastle Disease Viruses in Wild Birds
Source: PLoS One. 2016 Sep 14;11(9):e0162484. doi: 10.1371/journal.pone.0162484 (PMC5023329; doi:10.1371/journal.pone.0162484)
Supplement: S1 Table — (DOCX) [file pone.0162484.s001.docx]

**S1 Table. Demographic and correlated viral data of Rock Pigeons sampled in Atlanta, GA**.

The shedding status is presented with the name of each isolate; if birds were not shedding virus detectable by virus isolation, the results were marked with NEG. For age, U = unknown, HY = hatch-year, and AHY = after hatching year. Fat was scored on a scale of 0 (no visible fat) to 7 (bulging) in the wing pit. While weight-to-wing chord ratio was measured as the bird mass divided by the length of the longest un-flattened primary feather. Hemagglutination inhibition (HI) antibody titers equal or greater than 16 are considered for positive (values represent the reciprocal of the dilution). NP=not performed.

| Rock Pigeon ID | Isolate name | Age | Fat Score | Weight-to-wing chord ratio | HI Antibody Titer |
| --- | --- | --- | --- | --- | --- |
| 1 | NEG | U | 3 | 1.230 | <2 |
| 2 | NEG | U | 1 | 1.431 | <2 |
| 3 | NEG | HY | 0 | 0.904 | <2 |
| 4 | Rock Pigeon/USA/GA/5/2012 | HY | 1 | 1.125 | <2 |
| 5 | Rock Pigeon/USA/GA/9/2012 | HY | 2 | 1.309 | <2 |
| 6 | NEG | HY | 1 | 1.139 | NP |
| 7 | Rock Pigeon/USA/GA/8/2012 | HY | 1 | not determined | <2 |
| 8 | Rock Pigeon/USA/GA/7/2012 | HY | 3 | 1.056 | <2 |
| 9 | NEG | HY | 1 | 1.195 | 16 |
| 10 | NEG | HY | 2 | 1.271 | <2 |
| 11 | Rock Pigeon/USA/GA/4/2012 | HY | 1 | 1.110 | <2 |
| 12 | NEG | HY | 2 | 1.192 | <2 |
| 13 | NEG | HY | 1 | 1.212 | <2 |
| 14 | NEG | HY | 0 | 1.371 | <2 |
| 15 | NEG | HY | 3 | 1.208 | <2 |
| 16 | NEG | HY | 1 | 1.283 | <2 |
| 17 | NEG | HY | 2 | 1.220 | <2 |
| 18 | NEG | HY | 1 | 1.250 | <2 |
| 19 | Rock Pigeon/USA/GA/3/2012 | HY | 0 | 1.379 | <2 |
| 20 | NEG | HY | 1 | 1.009 | <2 |
| 21 | NEG | HY | 1 | 1.353 | <2 |
| 22 | NEG | HY | 1 | 1.410 | <2 |
| 23 | NEG | HY | 1 | 1.101 | <2 |
| 24 | NEG | HY | 1 | 1.250 | <2 |
| 25 | NEG | HY | 1 | 1.425 | <2 |
| 26 | NEG | HY | 1 | 1.034 | <2 |
| 27 | NEG | HY | 1 | 1.101 | NP |
| 28 | Rock Pigeon/USA/GA/6/2012 | HY | 0 | 1.151 | <2 |
| 29 | Rock Pigeon/USA/GA/1/2012 | HY | 1 | 1.080 | <2 |
| 30 | NEG | HY | 1 | 0.895 | <2 |
| 31 | NEG | HY | 3 | 0.787 | <2 |
| 32 | Rock Pigeon/USA/GA/2/2012 | HY | 2 | 1.123 | <2 |
| 33 | NEG | HY | 1 | 1.009 | <2 |
| 34 | NEG | HY | 1 | 1.146 | <2 |
| 35 | NEG | HY | 2 | 0.924 | <2 |
| 36 | NEG | HY | 1 | 1.429 | <2 |
| 37 | NEG | HY | 1 | 1.438 | NP |
| 38 | NEG | HY | 2 | 1.159 | <2 |
| 39 | NEG | HY | 2 | 1.216 | <2 |
| 40 | NEG | HY | 1 | 1.377 | <2 |
| 41 | NEG | HY | 1 | 1.357 | <2 |
| 42 | NEG | HY | 0 | 0.986 | <2 |
| 43 | NEG | HY | 2 | 1.1343 | <2 |
| 44 | NEG | HY | 1 | 1.395 | <2 |
| 45 | NEG | HY | 1 | 1.215 | <2 |
| 46 | NEG | HY | 3 | 1.360 | <2 |
| 47 | NEG | HY | 1 | 1.219 | <2 |
| 48 | NEG | HY | 1 | 1.391 | <2 |
| 49 | NEG | HY | 1 | 1.443 | NP |
| 50 | NEG | HY | 2 | 1.088 | <2 |
| 51 | NEG | AHY | 2 | 1.220 | <2 |
| 52 | NEG | AHY | 2 | 1.189 | <2 |
| 53 | NEG | AHY | 1 | 1.263 | <2 |
| 54 | NEG | AHY | 1 | 1.189 | <2 |
| 55 | NEG | AHY | 1 | 1.403 | <2 |
| 56 | NEG | AHY | 1 | 1.217 | <2 |
| 57 | NEG | AHY | 0 | 1.520 | NP |
| 58 | NEG | AHY | 1 | 1.499 | <2 |
| 59 | NEG | AHY | 1 | 1.271 | <2 |
| 60 | NEG | AHY | 1 | 1.624 | <2 |
| 61 | NEG | AHY | 1 | 1.312 | <2 |
| 62 | NEG | AHY | 2 | 1.478 | NP |
| 63 | NEG | AHY | 1 | 1.396 | 32 |
| 64 | NEG | AHY | 1 | 1.104 | <2 |
| 65 | NEG | AHY | 1 | 1.449 | <2 |
| 66 | NEG | AHY | 2 | 1.364 | <2 |
| 67 | NEG | AHY | 1 | 1.303 | <2 |
| 68 | NEG | AHY | 1 | 1.205 | <2 |
| 69 | NEG | AHY | 2 | 1.435 | <2 |
| 70 | NEG | AHY | 2 | 1.346 | 16 |
| 71 | NEG | AHY | 2 | 1.299 | NP |
| 72 | NEG | AHY | 2 | 1.473 | <2 |
| 73 | NEG | AHY | 1 | 1.457 | 8 |
| 74 | NEG | AHY | 0 | 1.409 | <2 |
| 75 | NEG | AHY | 1 | 1.580 | <2 |
| 76 | NEG | AHY | 1 | 1.473 | <2 |
| 77 | NEG | AHY | 2 | 1.456 | <2 |
| 78 | NEG | AHY | 1 | 1.354 | <2 |
