# Supplementary material for: Presence of Vaccine-Derived Newcastle Disease Viruses in Wild Birds
Source: PLoS One. 2016 Sep 14;11(9):e0162484. doi: 10.1371/journal.pone.0162484 (PMC5023329; doi:10.1371/journal.pone.0162484)
Supplement: S2 Table — (DOCX) [file pone.0162484.s002.docx]

**S2 Table. Rank sum scores for the variable Fat classified by the variable shedding**.

| Mean Rank Scores for Fat vs. Shedding | | | |
| --- | --- | --- | --- |
| Shedding Status | Individuals per Group (N) | Expected under H_0_ | Mean Fat Score |
| Negative | 36 | 828.0 | 23.291667 |
| Positive | 9 | 207.0 | 21.833333 |
