# Supplementary material for: Presence of Vaccine-Derived Newcastle Disease Viruses in Wild Birds
Source: PLoS One. 2016 Sep 14;11(9):e0162484. doi: 10.1371/journal.pone.0162484 (PMC5023329; doi:10.1371/journal.pone.0162484)
Supplement: S3 Table — Non-parametric test for ordinal values (HY birds only). (DOCX) [file pone.0162484.s003.docx]

**S3 Table. Kruskal-Wallis H test.** Non-parametric test for ordinal values (HY birds only).

| Statistic | Degrees of Freedom (*df*) | Test Statistic (Value) | Probability Value (P- value = p) |
| --- | --- | --- | --- |
| Kruskal-Wallis | 1 | 0.1117 | 0.7382 |
