# Supplementary material for: Presence of Vaccine-Derived Newcastle Disease Viruses in Wild Birds
Source: PLoS One. 2016 Sep 14;11(9):e0162484. doi: 10.1371/journal.pone.0162484 (PMC5023329; doi:10.1371/journal.pone.0162484)
Supplement: S4 Table — (DOCX) [file pone.0162484.s004.docx]

**S4 Table.** **Shapiro Wilk’s Test for Normality** for the variable weight-to-wing chord ratio (HY birds only).

| Statistic | N | Test Statistic (*W*) | Probability Value (P- value = p) |
| --- | --- | --- | --- |
| Shapiro-Wilk | 47 | 0.964707 | 0.1652 |
