# Supplementary material for: Presence of Vaccine-Derived Newcastle Disease Viruses in Wild Birds
Source: PLoS One. 2016 Sep 14;11(9):e0162484. doi: 10.1371/journal.pone.0162484 (PMC5023329; doi:10.1371/journal.pone.0162484)
Supplement: S5 Table — (DOCX) [file pone.0162484.s005.docx]

**S5 Table.** **GLM for the variable weight-to-wing chord** **ratio** (HY birds only), shedding versus non-shedding birds.

| GLM of weight-to-wing chord ratio | | | | | |
| --- | --- | --- | --- | --- | --- |
| Source | Degrees of Freedom (*df*) | Sum of Squares | Mean Square | Test Statistic (F) | P-value |
| Model | 1 | 0.00427863 | 0.00427863 | 0.17 | 0.6844 |
| Error | 42 | 1.07284233 | 0.02554387 |  | |
| Corrected | 43 | 1.07712096 |  |  | |
