# Supplementary material for: Presence of Vaccine-Derived Newcastle Disease Viruses in Wild Birds
Source: PLoS One. 2016 Sep 14;11(9):e0162484. doi: 10.1371/journal.pone.0162484 (PMC5023329; doi:10.1371/journal.pone.0162484)
Supplement: S6 Table — (DOCX) [file pone.0162484.s006.docx]

**S6 Table. Fisher’s Exact Test Full Model for comparing shedding vs. non-shedding by “age class” of Rock Pigeon sampled in Atlanta, GA.** Two-sided probability of Fisher’s Exact Test reflects the more conservative and increased error assumed in the algorithm to calculate the probability value.

| Statistic | Cell (1,1) Frequency (F) | Left-sided P-value | Right-sided P-value | Table Probability | Model Two-sided Probability |
| --- | --- | --- | --- | --- | --- |
| Fisher's Exact Test | 25 | 1.0000 | 0.0136 | 0.0136 | 0.0217 |
